# Supplementary material for: Plasma Proteomic Networks Reveal Shared Biology with Brain Linked to Alzheimer’s Disease Pathology
Source: medRxiv. 2026 Jun 2:2026.05.26.26353866. Preprint. [Version 1] doi: 10.64898/2026.05.26.26353866 (PMC13252471; doi:10.64898/2026.05.26.26353866)
Supplement: Supplementary file 1 [file NIHPP2026.05.26.26353866V1-supplement-1.pdf]

medRxiv preprint doi: https://doi.org/10.64898/2026.05.26.26353866; this version posted June 2, 2026. The copyright holder for this preprint (which was not certified by peer review) is the author/funder, who has granted medRxiv a license to display the preprint in perpetuity.
It is made available under a CC-BY 4.0 International license .

Supplemental Figure Legends

Supplemental Figure 1

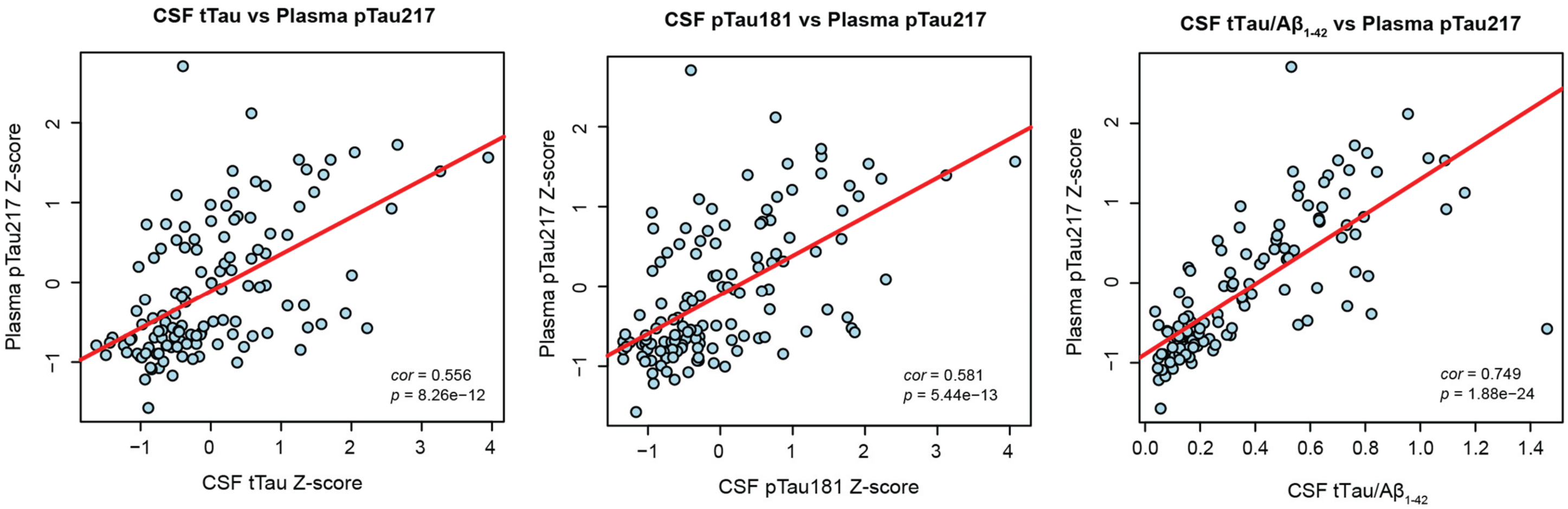

Supplemental Figure 1. Correlations Between Plasma pTau217 and CSF AD Biomarkers.

Scatter plots showing Pearson correlations between Z-scored plasma pTau217 and CSF AD biomarkers: CSF total tau (tTau; $r = 0.56$, $p = 8.3 \times 10^{-12}$), CSF phospho-tau181 (pTau181; $r = 0.58$, $p = 5.4 \times 10^{-13}$), and the CSF tTau/Aβ_{1-42} ratio ($r = 0.75$, $p = 1.9 \times 10^{-24}$). Statistical significance of correlations was assessed using Student's *t*-test.

medRxiv preprint doi: https://doi.org/10.64898/2026.05.26.26353866; this version posted June 2, 2026. The copyright holder for this preprint (which was not certified by peer review) is the author/funder, who has granted medRxiv a license to display the preprint in perpetuity.
It is made available under a CC-BY 4.0 International license .

Supplemental Figure 2

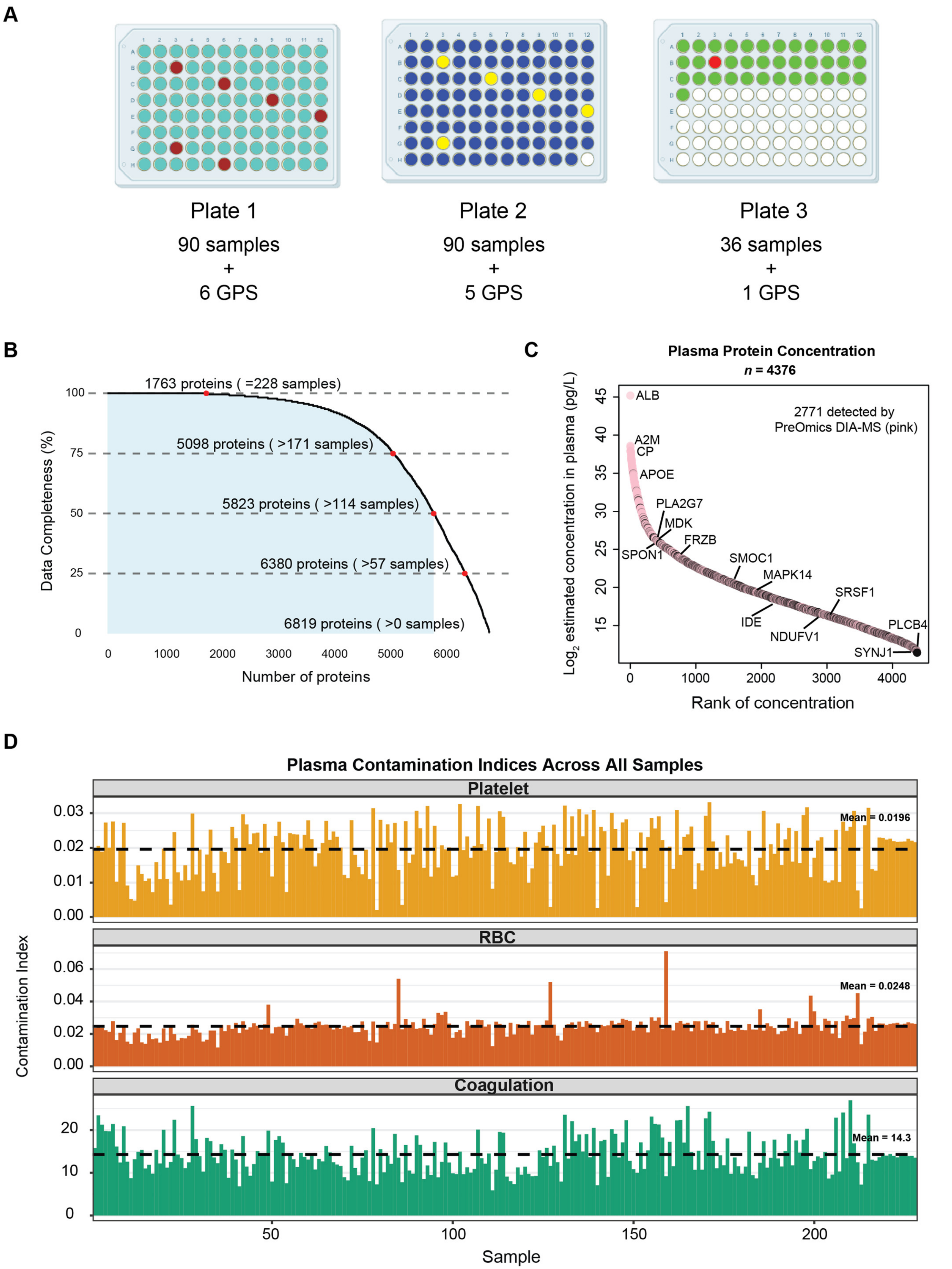

medRxiv preprint doi: https://doi.org/10.64898/2026.05.26.26353866; this version posted June 2, 2026. The copyright holder for this preprint (which was not certified by peer review) is the author/funder, who has granted medRxiv a license to display the preprint in perpetuity.
It is made available under a CC-BY 4.0 International license .

Supplemental Figure 2. Overview of Data Depth and Sample Quality.

A) Distribution of plasma samples (*n* = 216, including 2 duplicates) and Global Pooled Standards (GPS) samples (*n* = 12) across three 96-well plates. Plate 1 includes 90 samples (turquoise) and 6 GPS (brown); Plate 2 includes 90 samples (blue) and 5 GPS (yellow); Plate 3 includes 36 samples (green) and 1 GPS (red).
B) Protein measurement completeness across 228 samples: 1,763 proteins were quantified in all 228 samples (no missing values, i.e., 100% completeness); 5,823 proteins were detected in more than half of the samples and were retained for downstream analysis.
C) Of the 4,376 plasma proteins with estimated concentrations reported in the Human Protein Atlas database (47), 2,771 were detected using our PreOmics DIA-MS workflow. All 4,376 proteins were ranked by their estimated concentrations (log_2-transformed, pg/L), with proteins detected by DIA-MS highlighted in pink and those not detected shown in black. Protein abundances span approximately ten orders of magnitude, and low-abundance proteins such as SYNJ1 and PLCB4 were successfully detected.
D) Potential contamination from platelets, erythrocytes (RBC), and coagulation-related proteins was evaluated using Baize software on raw, unnormalized protein abundance data from all 228 plasma samples, including technical duplicates. Each bar represents one sample, and dashed horizontal lines indicate the cohort mean contamination index for each category.

medRxiv preprint doi: https://doi.org/10.64898/2026.05.26.26353866; this version posted June 2, 2026. The copyright holder for this preprint (which was not certified by peer review) is the author/funder, who has granted medRxiv a license to display the preprint in perpetuity.
It is made available under a CC-BY 4.0 International license .

Supplemental Figure 3

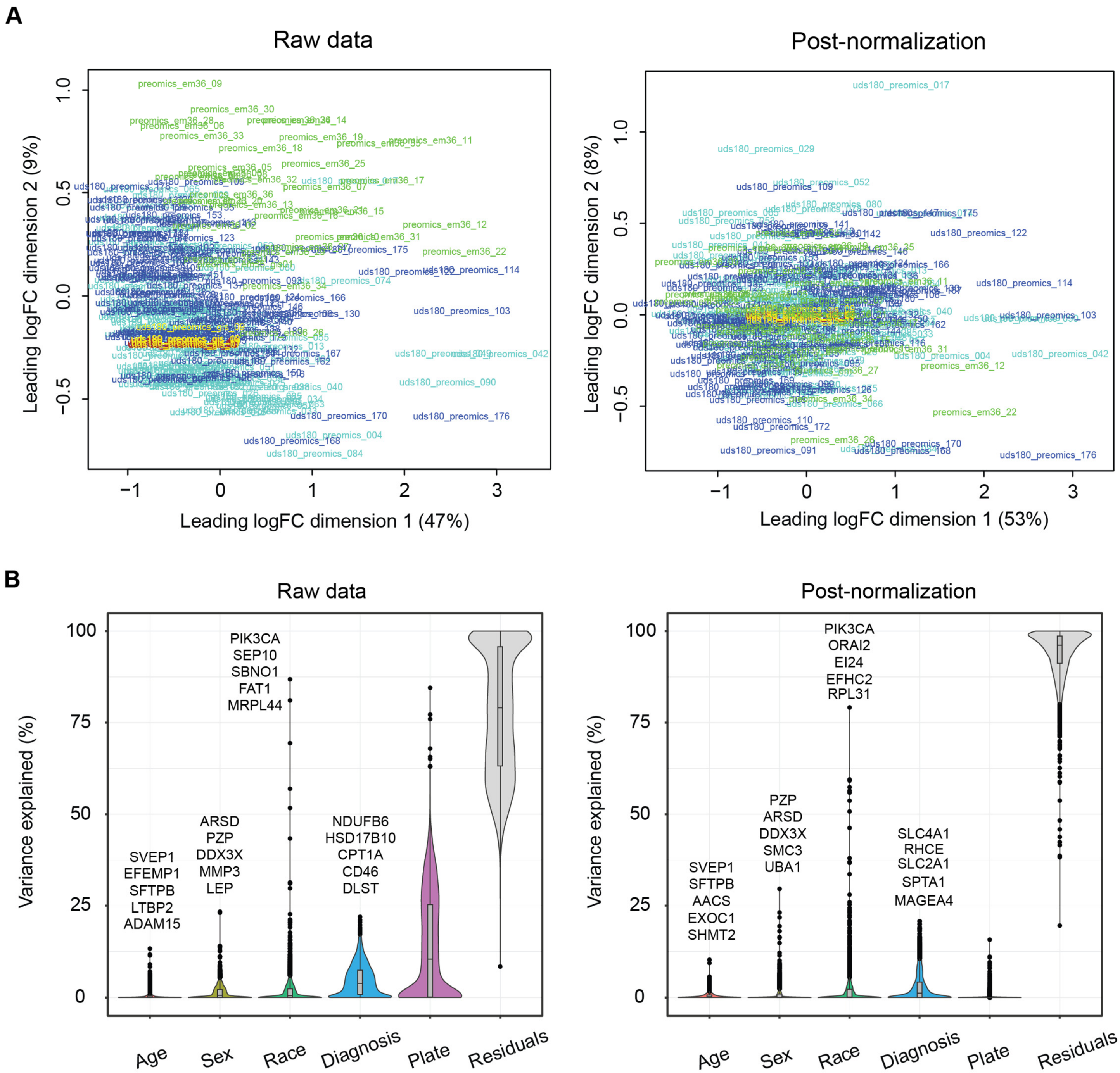

Supplemental Figure 3. TAMPOR Normalization Effectively Removes Plate-Related Variance While Preserving Biological Signals in Plasma DIA-MS Data.

A) Protein abundance data from 216 plasma samples and 12 global pooled standards (GPS) were normalized using the Tunable Approach for Median Polish of Ratios (TAMPOR). Multidimensional scaling (MDS) plots display sample clustering before (left) and after (right) normalization. In the raw data, samples cluster by plate—Plate 1 (blue), Plate 2 (turquoise), Plate 3 (green), and GPS samples (brown, yellow, or red)—indicating strong plate effects. After TAMPOR normalization, samples cluster tightly regardless of plate, demonstrating effective removal of plate-related variance.

B) Variance partition analysis before (left) and after (right) TAMPOR normalization. The y-axis shows the percentage of variance explained by each factor (age, sex, race, diagnosis, plate, and residuals). After normalization, variance attributed to plate is markedly reduced, while biological variance is preserved. The top five proteins most influenced by age, sex, race, and diagnosis are labeled.

medRxiv preprint doi: https://doi.org/10.64898/2026.05.26.26353866; this version posted June 2, 2026. The copyright holder for this preprint (which was not certified by peer review) is the author/funder, who has granted medRxiv a license to display the preprint in perpetuity.
It is made available under a CC-BY 4.0 International license .

Supplemental Figure 4

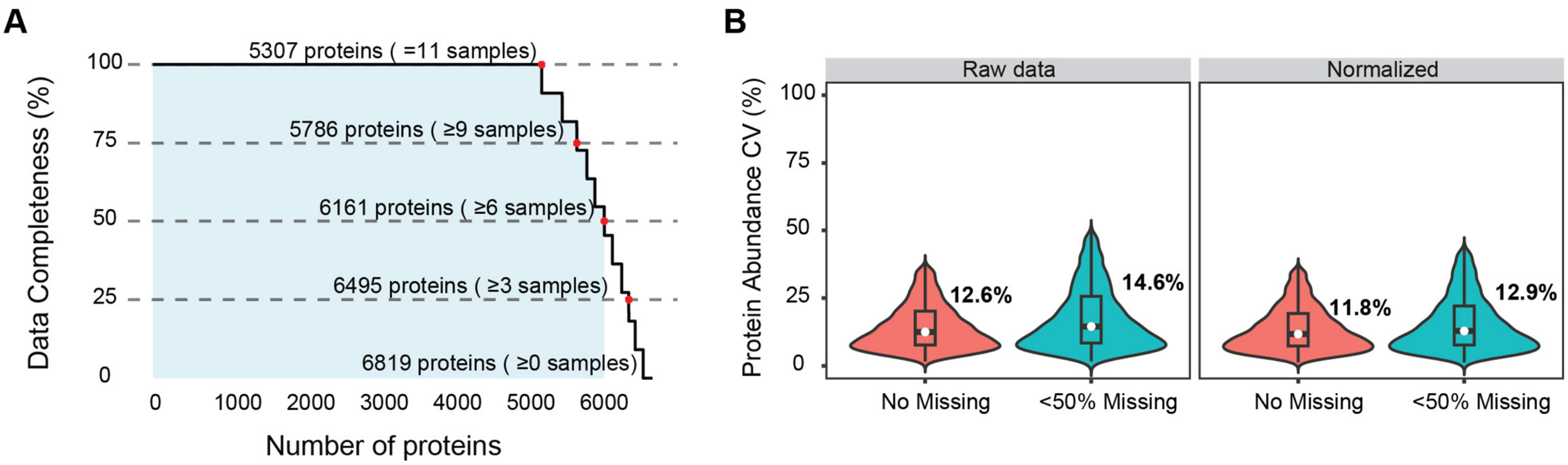

Supplemental Figure 4. High Technical Reproducibility and Low Post-Normalization Variance across Global Pooled Standard (GPS) Samples.

A) Data completeness across 11 GPS samples. A total of 5,307 proteins were quantified in all GPS samples (no missing values), and 6,161 proteins were detected in more than half of the samples (<50% missing).
B) Violin plots showing protein abundance variability (coefficient of variation, CV) across GPS before and after normalization (instrument variance). Proteins quantified without missing values (orange) and those detected in at least 6 of the 11 GPS (green) are shown. Median CVs are labeled for each plot. For visualization purposes, CV values outside the range of mean ± 2 standard deviations (~5% for each group) are not shown in the violin plots. All statistics are calculated using the full dataset, including all proteins.

medRxiv preprint doi: https://doi.org/10.64898/2026.05.26.26353866; this version posted June 2, 2026. The copyright holder for this preprint (which was not certified by peer review) is the author/funder, who has granted medRxiv a license to display the preprint in perpetuity.
It is made available under a CC-BY 4.0 International license .

Supplemental Figure 5

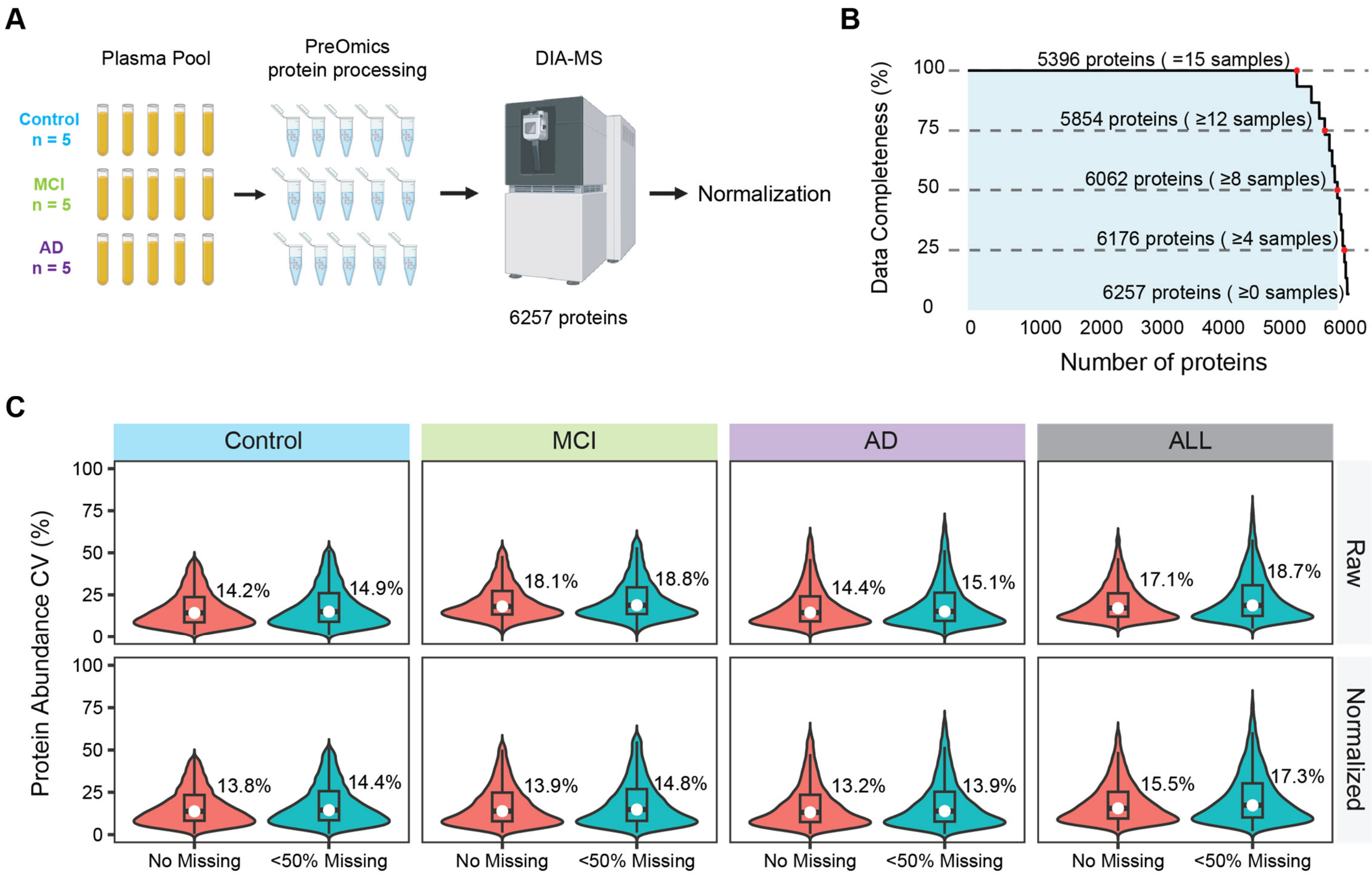

Supplemental Figure 5. Microbead Enrichment Workflow Demonstrates High Proteome Coverage and Reproducibility.

A) Workflow of 15 pooled plasma samples (5 Control, 5 MCI, 5 AD) processed the same way as cohort samples using the PreOmics DIA-MS pipeline, identifying 6,257 proteins.
B) Data completeness across the 15 pooled plasma samples, with 5,396 proteins quantified in all samples (no missing values) and 6,062 detected in more than half of the samples.
C) Violin plots showing distributions of protein abundance variability (coefficient of variation, CV) before and after normalization across pooled plasma samples (technical variance). Proteins quantified without missing values are shown in orange, and those detected in at least 8 of the 15 pooled plasma samples are shown in green. Median CVs are labeled for each plot. For visualization purposes, CV values outside the range of mean ± 2 standard deviations (~1%-5% for each group) are not shown in the violin plots. All statistics are calculated using the full dataset, including all proteins.

medRxiv preprint doi: https://doi.org/10.64898/2026.05.26.26353866; this version posted June 2, 2026. The copyright holder for this preprint (which was not certified by peer review) is the author/funder, who has granted medRxiv a license to display the preprint in perpetuity.
It is made available under a CC-BY 4.0 International license .

Supplemental Figure 6

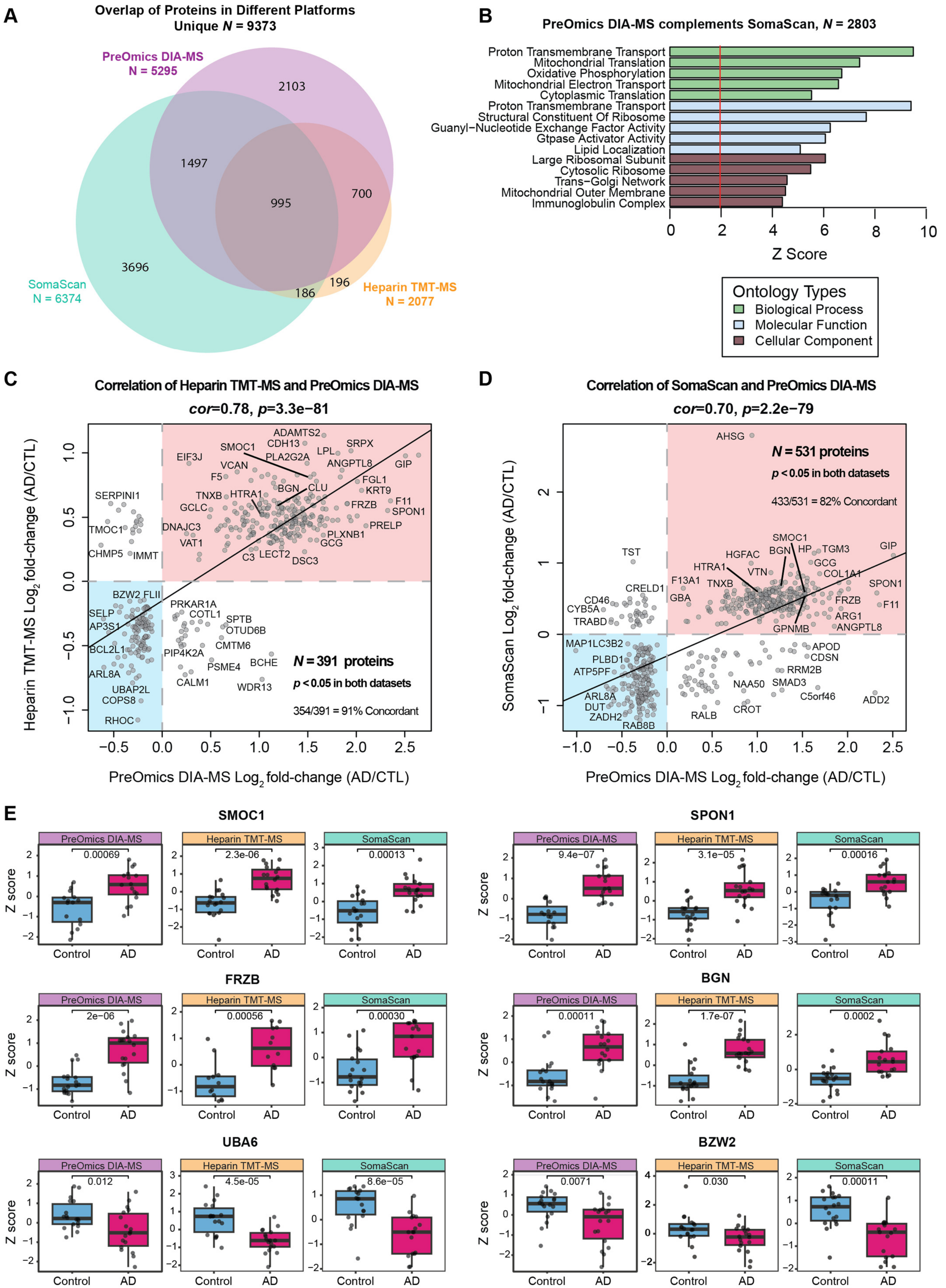

medRxiv preprint doi: https://doi.org/10.64898/2026.05.26.26353866; this version posted June 2, 2026. The copyright holder for this preprint (which was not certified by peer review) is the author/funder, who has granted medRxiv a license to display the preprint in perpetuity.
It is made available under a CC-BY 4.0 International license .

Supplemental Figure 6. Cross-Platform Validation of AD Plasma Proteomics Across Mass Spectrometry and Aptamer-Based Assays.

A) Overlap of plasma proteins quantified across three independent platforms using overlapping samples: microbead-enriched DIA-MS (PreOmics DIA-MS, $N = 5{,}295$ proteins; Control = 18, AD = 18), heparin-enriched TMT-MS (Heparin TMT-MS, $N = 2{,}077$ proteins; Control = 18, AD = 18), and aptamer-based assays (SomaScan, $N = 6{,}374$ proteins; Control = 18, AD = 17). Only proteins detected in at least 18 samples per platform were included.
B) Gene Ontology (GO) enrichment of 2,803 proteins uniquely identified by PreOmics DIA-MS relative to SomaScan. These proteins were enriched for mitochondrial and translational processes, including mitochondrial translation and mitochondrial outer membrane, highlighting complementary coverage of intracellular biology beyond aptamer-based assays.
C–D) Pairwise correlations (Pearson correlation) of $\log_2$ fold-change (AD vs. Control) between PreOmics DIA-MS and Heparin TMT-MS (**C**, $r = 0.78$, $p = 3.3 \times 10^{-81}$; $N = 391$ proteins, 91% concordant) and between PreOmics DIA-MS and SomaScan (**D**, $r = 0.70$, $p = 2.2 \times 10^{-79}$; $N = 531$ proteins, 82% concordant). Only proteins overlapping and significantly altered ($p < 0.05$) in both datasets were included.
E) Boxplots showing consistent protein abundance changes in AD across all three platforms for SMOC1, SPON1, FRZB, BGN, UBA6, and BZW2. Statistical significance was determined using Student's *t*-test.
